# Longitudinal patterns of companion animals in families with children during the COVID-19 pandemic: Findings from the Adolescent Brain Cognitive Development (ABCD) Study^®^

**DOI:** 10.3389/fvets.2024.1364718

**Published:** 2024-04-24

**Authors:** Erin K. King, Seana Dowling-Guyer, Emily McCobb, Megan K. Mueller

**Affiliations:** ^1^Cummings School of Veterinary Medicine, Tufts University, North Grafton, MA, United States; ^2^Tisch College of Civic Life, Tufts University, Medford, MA, United States

**Keywords:** companion animal, pet ownership, COVID-19, demographics, pandemic (COVID19)

## Abstract

Pet acquisition purportedly increased during the COVID-19 pandemic with individuals acquiring pets during periods of social isolation. Families with children experienced unique challenges during the COVID-19 pandemic, balancing childcare, remote schooling, and other needs and therefore patterns of pet acquisition and loss may differ from the broader population. The goal of this study was to understand patterns of pet ownership within families with adolescents during the pandemic to help identify areas for improved support and programmatic recommendations. Using self-reported survey data from a sample of 7,590 American adolescents from the Adolescent Brain Cognitive Development (ABCD) Study^®^ COVID Survey, we found no evidence for large-scale changes in pet acquisition or relinquishment during the first year of the pandemic for families with adolescents in the U.S. Future research should explore the effects of pet acquisition and pet loss on families with adolescents and what resources are needed to support pet ownership during stressors such as the COVID-19 pandemic.

## Introduction

During the COVID-19 pandemic, pet acquisition purportedly increased with major media attention focusing on “pandemic pets” or pets acquired during the pandemic lockdown [e.g., (1)]. With the shift to remote, work-from-home schedules, public desire for pet adoption and fostering reportedly increased in the United States and around the globe (2, 3). However, other data on pet ownership rates and adoption trends suggest that pet ownership rates did *not* increase over the pandemic (4). For example, Powell et al. (5) found the proportion of animals adopted from a sample of shelters did not increase in 2020 as compared to 2019 and Shelters Animals Count reported an estimated 1,381,595 adoptions in 2019 compared to 1,195,590 animals in 2021 (6). This ambiguity highlights the need for more data examining pet ownership patterns during and after the pandemic, in order to fully understand how the pandemic has impacted pet acquisition and ownership trends in the United States. Furthermore, families with children experienced unique challenges during the pandemic, balancing childcare, remote schooling, and other needs. It is plausible that patterns of pet acquisition and loss in households with youth may differ from the broader population. In order to identify areas for improved support for families with children and pets during stressors such as the COVID-19 pandemic, we need to understand patterns of pet ownership during this time period in families with adolescents in the home.

Companion animals are key members of the family, with about 66% of households owning a pet in the United States (7). Households with children have increased odds of having a pet compared to homes without a child (8, 9) and the American Veterinary Medical Association (AVMA) reported that homes with married couples and children were more likely to acquire a pet during the COVID-19 pandemic (10). Pets often play a prominent role in the lives of children and adolescents, and are an important component of how youth cope with stress, as many youth report turning to their pets for support and emotional comfort when experiencing stress (11). During the COVID-19 pandemic when youth were experiencing anxiety, depression, and other stressors (12), a pet relationship might have been particularly critical. For teenagers in particular, social relationships are a crucial component of this developmental period (13). In the absence of in-person social interactions, such as during the pandemic, pets may have provided another source of social and emotional support. Prior research on companion animal relationships has found that youth-pet relationships are associated with adaptive social coping behaviors (14), prosocial behaviors such as empathy (15, 16), and socio-emotional skills (17). In fact, interacting with a pet is one of the most prevalent ways that teenagers reported coping with stressors during the pandemic (8, 9), and some research on pet ownership in COVID more broadly suggested that pets were an important component of well-being during this time (18, 19). It could also be that the physical comfort provided by pets may have been particularly important during the COVID-19-pandemic, where physical touch was limited (20).

However, while adding a pet to a family with adolescents may have positive effects such as reduced social isolation, having a companion animal can also add to caregiving responsibilities. Challenges to pet ownership such as access to veterinary care and pet supplies, cost, and balancing responsibilities were exacerbated during the pandemic (21, 22). For families managing a lack of childcare and in-person schooling for their children, having or acquiring a pet might have been a significant burden. Increased uncertainty from the pandemic may have impacted homes with children more than the general population because sudden changes in available resources and potential risk of hardship creates stress in families, especially those with children (23–25). It is important to understand how pet ownership trends may vary during times of stress and routine disruption, particularly in homes with youth. Understanding these patterns may help inform future research on both the benefits of pet relationships during both the pandemic and other stressful events, as well as challenges and barriers to acquiring or keeping a pet. For example, future work should explore the role of access to affordable veterinary care, food security, housing security, and other family and community-level factors that can impact pet ownership for families. Ultimately, understanding these relationships will inform proactive interventions to support families and their pets.

Because of the important role pets can play in the lives of families with adolescents, as well as the complexities of how pets fit into the family system during the unique period of COVID-19, there is a need to understand patterns of pet acquisition and loss during this time using longitudinal data. Although the COVID-19 pandemic has moved into a more stable state, exploring patterns of pet acquisition during this time period is critical for two reasons. First, pet ownership trends during the COVID-19 pandemic among households with adolescents are particularly important to explore because lockdowns, school closures, and social distancing guidelines increased social isolation (26) and adolescents in particular are at risk for ongoing negative impacts from the pandemic due to the consequences of this isolation (27). Although the pandemic is not currently in an acute stage, families that acquired or lost pets during this time period may still be in need of support to optimize the human-animal bond. Secondly, it is useful to leverage data from the COVID-19 to assess patterns of pet acquisition and loss during times of stressors more broadly in order to be more adequately prepared in the future to help families manage both the benefits and challenges of having a pet during a challenging time. Therefore, it is important to understand ownership trends during the pandemic and if the pandemic interrupted or encouraged pet ownership in households with children. The goal of this study was to understand patterns of pet ownership within families with adolescents in the United States during the initial stages of the COVID-19 (2020–2021) pandemic to help identify substantial increases and decreases in pet ownership rates that would indicate the need to explore how to best support families with adolescents as we move into the post-COVID era.

## Methods

### Participants and procedure

This research used data from the Adolescent Brain Cognitive Development (ABCD) Study®, a longitudinal study of brain development and youth health outcomes in the United States. The ABCD Study collects annual or bi-annual physical, cognitive, social, emotional, environmental, behavioral, and academic assessments of youth over a 10-year period. A baseline cohort of 11,878 youth enrolled at 9–10 years of age, along with their parents/guardians and participants are included in the study until they are 19 or 20 years old. Initial participants were recruited to participate in the ABCD Study between July 2016 and August 2018. Recruitment areas were determined by locations of 21 study sites (catchment areas), which closely matched the sociodemographic composition of the United States population. Within the catchment areas, participants were recruited through school-based probability sampling, where schools within each area were coded based on geographic location and sociodemographic characteristics using data from the National Center for Education Statistics, and converted into databases that were used to generate lists for random selection (28). The sample target uses a slight oversampling of racial/ethnic minority youth to ensure a demographically diverse sample [for more information see https://abcdstudy.org/scientists/ and Garavan et al. (28)]. The overall study procedures were approved by each local ethics committee of the relevant institutions per the National Institutes of Health (NIH) human subjects research guidelines.

This study involved secondary analysis of a subset of de-identified survey data (*N* = 9,048) from the ABCD COVID Rapid Response Research (RRR) Surveys (DOI 10.15154/1520584 and 10.15154/1522601). Surveys asking about the impact of the pandemic on participants’ lives were administered electronically six times; May 2020, June 2020, August 2020, October 2020, December 2020, and March 2021. Parent-reported demographic data were derived from the ABCD Annual Curated Release 3.0 (DOI 10.15154/1519007). For this analysis, we used data from six time points of the RRR survey. Approximately 8.6% of the sample (*n* = 855) was missing data for all the key variables of interest, including pet ownership and therefore were removed from the sample for analysis. In addition, due to our primary variable of interest (pet ownership) being a family-level characteristic, we used only one sibling per family. For multi-child families, we used data from the adolescent who had the most waves of data. If both/all children had the same number of waves of data, we randomly selected one sibling. The final analytic sample was *N* = 7,590 and demographic information can be found in Table 1.

**Table 1:** Demographic information of youth in sample.

	Total sample (*N* = 7,590)
Mean age time point 1 (years)	12.4 (0.86)
Range age time point 1 (years)	10.6–14.7
Sex at birth*	*n* (%)
Female	3,776 (49.7%)
Male	3,814 (50.3%)
Race/Ethnicity**	
White	5,891 (77.6%)
Black	1,350 (17.8%)
Hispanic	1,513 (20.2%)
Asian	551 (7.3%)
Other Race	522 (6.9%)
Indigenous	248 (3.3%)
Parental employment status***	
Employed	5,418 (71.4%)
Unemployed	2015 (26.5%)

*No participants reported “other.” **Racial/ethnic identity groups are not mutually exclusive. ***N missing = 157.

### Measures

#### Demographics

Youth participants reported their age (recorded in months, re-coded to years) and youth sex at birth (female, male, other, not reported). Participants’ parents/guardians were also asked to identify their child’s racial and ethnic identity. Due to small sample sizes in each category, racial/ethnic identity was combined into non-mutually exclusive analytic categories of White, Black/African American, Asian (Asian Indian, Chinese, Japanese, Vietnamese, Korean, Filipino), Indigenous (American Indian, Native American, Alaska Native), Other Racial Identity, and Hispanic/Latinx. Parents/guardians reported their employment status, which was recoded into “employed” (working now: full time part time, maternity leave, sick leave) and “unemployed” (temporarily laid off, looking for work, unemployed not looking for work, retired, disabled: permanently or temporarily, stay at home parent, student).

#### Pet ownership

Pet ownership was measured by asking youth participants if they had a pet, and if so, what kind of pet/s did they have [dog, cat, horse, fish, small animal (e.g., rabbit, hamster, bird), or other]. Having a pet in the home was assessed at each of the six time points, which was then used to calculate variables capturing transitions in and out of pet ownership across time points. We also calculated transitions in the two most prevalent types of pets, dogs and cats. Pet acquisition was characterized by a participant selecting “no” to owning a pet and changing to “yes” in later time points. Pet loss was operationalized as a participant selecting “yes” to owning a pet at one time point and then changing to “no” at a subsequent time point.

### Data analysis

All analyses were performed in SPSS Version 26 and Microsoft Excel. Descriptive statistics and frequencies are reported for demographics characteristics. We also descriptively explored patterns of pets in households over six time points and assessed if patterns differed by species of pet. In addition, we used a chi-square analysis to assess if demographic variables were associated with pet acquisition/loss during the study period (with *p* < 0.05 used as the threshold for significance).

## Results

**Figure 1:**
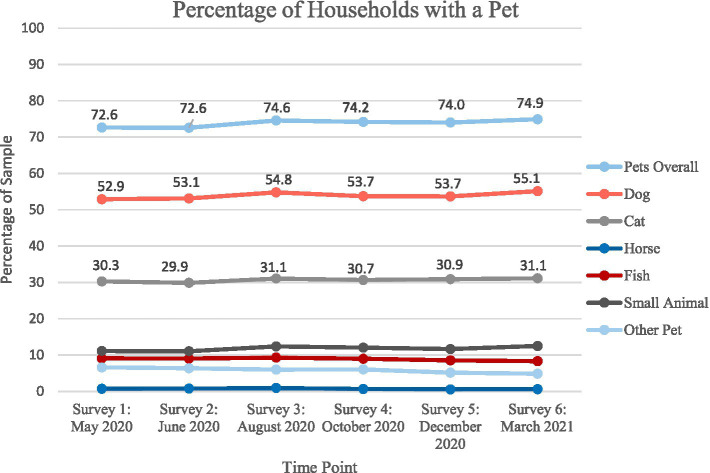
Percentage of households with a pet: May 2020 to March 2021.

In May of 2020, approximately 73% of participants reported that they had at least one pet. Figure 1 shows pet ownership trends over 1 year, with a slight increase in pet ownership rates to 75% by May 2021. Percentages of households with each species can also be found in Figure 1. Dogs were the most common species in our sample of households with adolescents, followed by cats and small animals, fish, other pets, and horses. Species did not appear to substantially impact the increase or decrease of pet ownership across timepoints.

**Figure 2:**
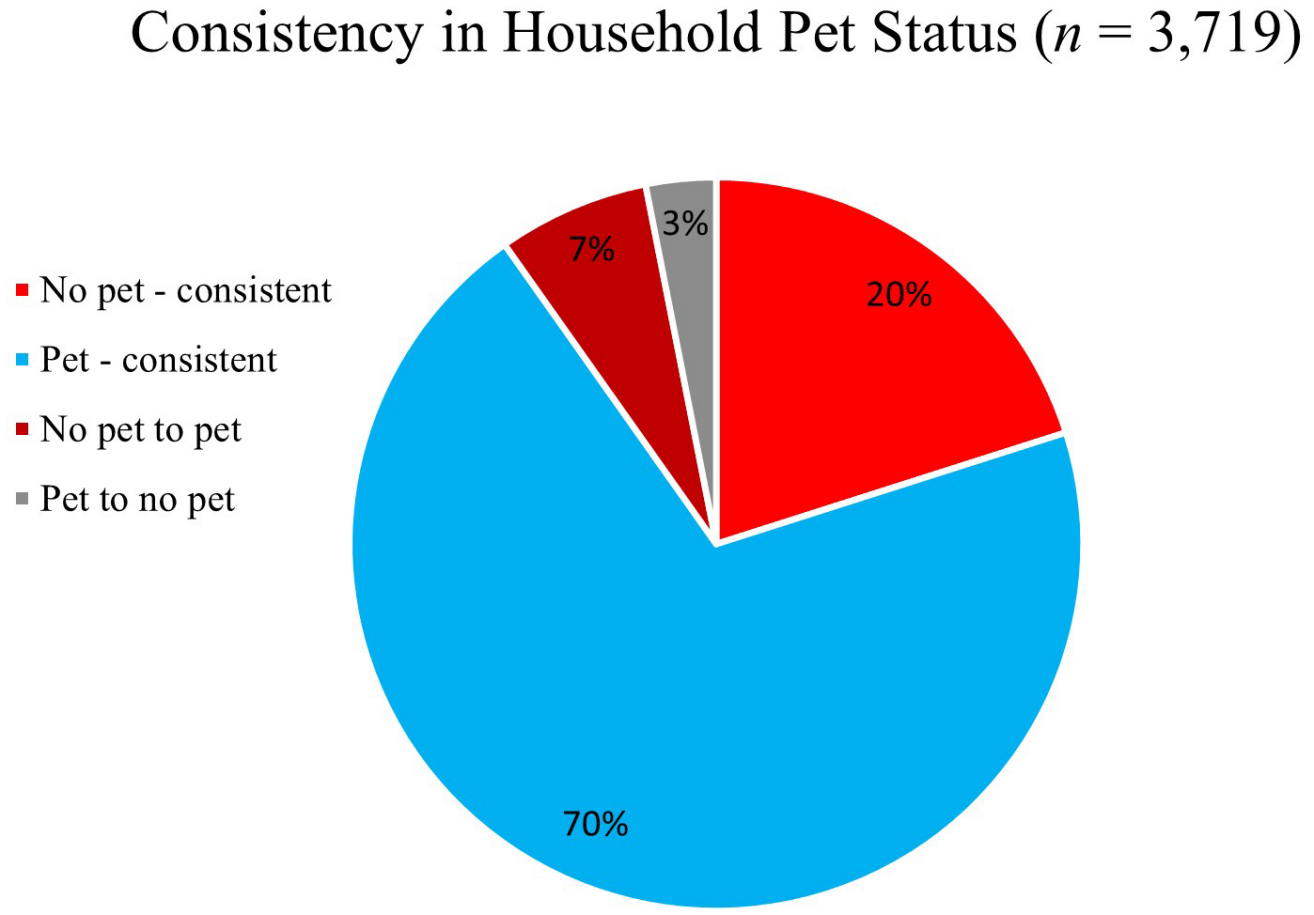
Pet ownership status across all time points.

**Figure 3:**
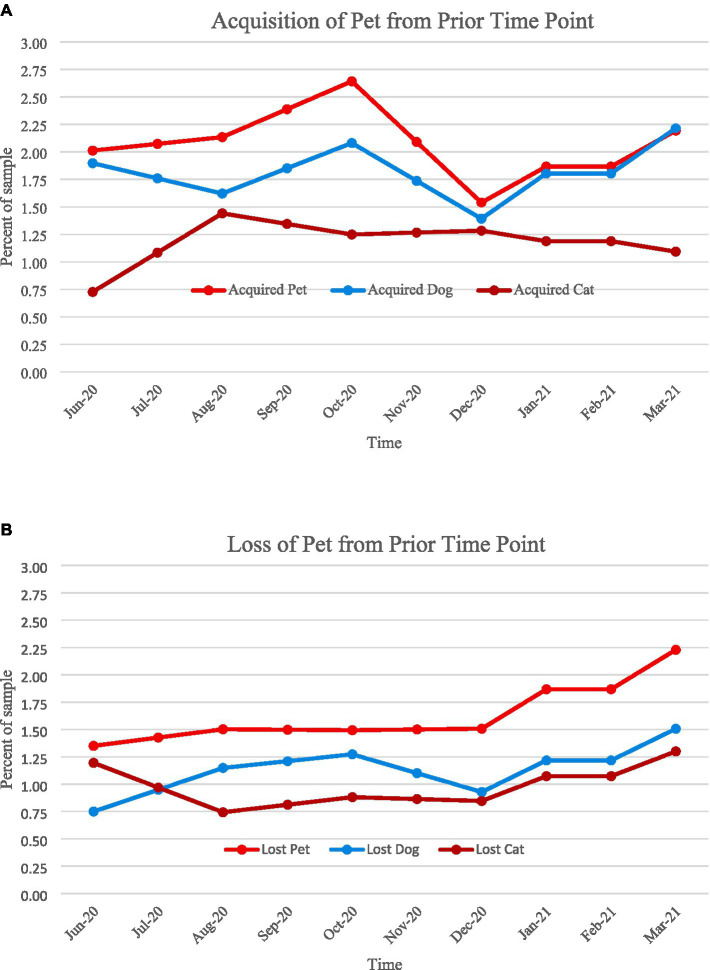
Percentage of households with a change in pet ownership status. **(A)**: Acquisition of a pet from prior time point, **(B)**: Loss of a pet from prior time point.

In order to understand trends in whether households were acquiring new pets or losing pets (e.g., due to death or rehoming), we analyzed the change in pet ownership status across time points using a subsample of participants who had at least four of the six data points (*n* = 3,719). As shown in Figure 2, we did not find evidence for substantial changes in pet acquisition or relinquishment during the first year of the pandemic in our sample. In fact, only 7% of participants went from non-pet owners to pet owners. Seventy percent of participants consistently owned an animal throughout multiple time points, and about 20% of participants consistently never owned an animal. Patterns regarding the percentage of participants who acquired or lost a pet during the pandemic can be seen in Figure 3. There was a small increase in pet acquisition during fall 2020, and a small increase in pet loss during spring 2021, though these are not substantial differences.

Chi-square analyses indicated that sex at birth, employment status, and racial/ethnic identity were associated with whether a family was a consistent pet owner, a consistent no pet owner, acquired a pet, or lost a pet. However, these differences were primarily related to consistency in having a pet, with participants who identified as female, White, employed, and Indigenous being more likely to consistently have a pet. There were fewer clear patterns among demographic variables predicting acquisition or loss of a pet, though Black participants appeared to have higher rates of acquisition than non-Black participants (see Table 2 for full results).

**Table 2:** Demographic variables predicting consistency in pet ownership status for participants with at least four time points of data (*n* = 3,719).

Demographic characteristic	Consistency in pet ownership status						
	Consistent pet owner % (n)	Consistent no pet % (n)	Acquired a pet % (n)	Lost a pet % (n)	χ^2^	df	*p*-value
Female	72.5% (1422)	17.4% (340)	6.5% (126)	3.3% (64)	19.16	3	**<0.001**
Male	67.2% (1187)	23.0% (406)	6.9% (122)	2.9% (52)			
Employed	72.1% (1939)	18.7% (502)	6.1% (165)	3.2% (85)	22.29	3	**<0.001**
Unemployed	64.3% (619)	24.5% (236)	8.1% (78)	3.1% (30)			
Race/Ethnicity*							
White	75.7% (2322)	15.2% (465)	6.1% (187)	3.0% (93)	295.17	3	**<0.001**
Non-White	44.0% (287)	43.1% (281)	9.4% (61)	3.5% (23)			
Black	44.6% (219)	40.9% (201)	11.0% (54)	3.5% (17)	191.68	3	**<0.001**
Non-Black	74.0% (2390)	16.9% (545)	6.0% (194)	3.1% (99)			
Asian	60.5% (207)	29.8% (102)	7.0% (24)	2.6% (9)	23.24	3	**<0.001**
Non-Asian	71.1% (2402)	19.1% (644)	6.6% (224)	3.2% (107)			
Indigenous	79.1% (72)	14.3% (13)	4.4% (4)	2.2% (2)	3.60	3	0.309
Non-indigenous	69.9% (2537)	20.2% (733)	6.7% (244)	3.1% (114)			
Other race	57.5% (119)	30.4% (63)	8.2% (17)	3.9% (8)	17.95	3	**<0.001**
Non-other race	70.9% (2490)	19.4% (683)	6.6% (231)	3.1% (108)			
Hispanic	63.1% (384)	25.3% (154)	8.4% (51)	3.3% (20)	18.16	3	**<0.001**
Non-Hispanic	71.5% (2198)	19.1% (588)	6.3% (193)	3.1% (96)			

*Groups are not mutually exclusive. Reference category is participants who do not identify as that category.

## Discussion

The aim of this study was to understand patterns of pet ownership within families with adolescents during the initial stages of the COVID-19 pandemic (spring 2020 through spring 2021). Results show consistent trends of pet ownership status, with a slight increase from 73 to 75% of the sample owning pets. We found that pet ownership rates in homes with adolescents was higher than the U.S. national average (~66%; APPA, 2023), which is consistent with findings from other studies showing that pet ownership tends to be higher in families with children (8, 9, 29). Similarly, we found that dogs are the most common household pet, which is often mirrored in national studies on pet populations (7).

We did not find strong evidence to support a large scale “pandemic pet” boom in our sample of households with children, though there was a small increase in pet acquisition in fall 2020, rising from 1.9% of the sample acquiring a pet in June 2020 to 2.7% in October 2020. Our findings indicate somewhat lower levels of pet acquisition compared to a 2021 ASPCA report suggesting that one in five households (about 19% of respondents) in the United States acquired a dog or cat during March 2020 to May 2021 (30). This difference in pet acquisition between families with adolescents as compared to the overall population in the United States could be for a variety of reasons. First, it could be that households with adolescents were less socially isolated due to the close proximity of other family members, therefore these households did not acquire pets to help ease social isolation. While research on the association between pets and loneliness has produced mixed findings [e.g., (31, 32)], there is little research on the specific reasons for pet acquisition and if these reasons vary by household composition. For example, individuals living alone or older adults with an increased risk of social isolation in some cases were more likely to have acquired a pet during the pandemic (33), but more research is needed on the motivations behind pet acquisition decisions. Second, Packer et al. (34) found that in the United Kingdom, 87% of respondents were motivated to acquire a puppy during the pandemic because they had more time to care for the dog. It could be that in households with adolescents, the pandemic created challenging transitions to home school and remote work, which added stress (35) and limited free time in the family. Due to this increased stress, households with adolescents may not have felt like they had capacity to add pets to the home. Additionally, the pandemic often made caregiving roles more burdensome so families without pets could have hesitated to add more responsibilities during this challenging time.

Pet loss was also not substantial over the course of the first year of the pandemic in homes with adolescents, with only a slight increase in pet loss in spring 2021 (from 1.5% in December 2020 to 2.2% in March 2021). These findings are similar to the ASPCA report (2021) that found 90% of households that acquired a dog and 85% of households that acquired a cat still had that animal in the home. However, we did find that demographic variables were predictive of overall prevalence of pet ownership stability, such that female, White, and employed participants were more likely to consistently have a pet, and Black participants were less likely to consistently own a pet, but also more likely to have acquired a pet. These findings point to the potential co-occurrence of systematic inequalities that were prevalent across many aspects of the pandemic that may intersect with pet ownership. Overall, the sample sizes of participants who acquired or lost a pet were generally too small to make generalizations; the majority of the sample across demographic variables were consistent in whether or not they had a pet across the time points.

While we were able to assess if there was a change in pet ownership status, a major limitation to this study is that we do not know the reason someone was no longer a pet owner. It could be that the pet was rehomed, relinquished to a shelter, or passed away. Future research should explore reasons behind pet loss during the COVID-19 pandemic and other challenging times, in order to understand what resources could support pet owners keeping their companion animals in the home.

## Limitations and conclusion

This research leveraged a large, longitudinal dataset that began data collection relatively soon after the beginning of the pandemic (May 2020), allowing for tracking of pet ownership trends longitudinally during 2020–2021. However, there are some limitations to the sample, including a lack of racial and ethnic diversity and lack of information on gender identity (vs. sex at birth). Future studies should work to increase the representative nature of research on companion animal trends to accurately reflect national trends. Another limitation is the lack of information and reasoning for pet acquisition and pet loss, as this is often affected by demographic variables (36). Motivations and decisions regarding pet acquisition and relinquishment are frequently complex (37–39) and need further investigation in order to provide support to families. Additionally, because data only captured overall household pet acquisition and loss, changes to multi-pet households in which families added or lost a pet while still maintaining other pets could not be assessed. Finally, this dataset only included households with early adolescents aged 10–14 years. Future studies should investigate comparisons between households with and without children of all ages to understand broader pet ownership trends and how different types of family composition may impact reasons for acquiring or losing a pet during times of increased stress and disruption of normal life due to things like a pandemic.

Despite the limitations of this research, this study is the first to use a large, longitudinal dataset to understand patterns of pet ownership in households with youth during the COVID-19 pandemic in the United States. We did not find evidence for substantial changes in pet acquisition or relinquishment during the first year of the pandemic for families with adolescents in the U.S. Future research should explore reasons that families with children of all ages acquire and lose pets during challenging situations and identify support to help families maintain healthy relationships with their pets. As the COVID-19 pandemic begins to dissipate in the United States, more research on companion animals will be needed in the future to understand the lasting impacts of the pandemic on both humans and pets.

## Data availability statement

The datasets presented in this study can be found in online repositories. The names of the repository/repositories and accession number(s) can be found in the article/supplementary material.

## Ethics statement

This paper is secondary analysis of de-identified data. Informed consent was obtained from the individual(s) who participated in the original ABCD Study.

## Author contributions

EK: Data curation, Investigation, Writing – original draft, Writing – review & editing, Project administration. SD-G: Conceptualization, Writing – review & editing, Investigation. EM: Writing – review & editing, Conceptualization, Investigation. MM: Conceptualization, Formal analysis, Investigation, Visualization, Writing – review & editing, Funding acquisition, Methodology, Project administration, Resources, Supervision.
